# Nuclear Genome Sequence and Gene Expression of an Intracellular Fungal Endophyte Stimulating the Growth of Cranberry Plants

**DOI:** 10.3390/jof9010126

**Published:** 2023-01-16

**Authors:** Bhagya C. Thimmappa, Lila Naouelle Salhi, Lise Forget, Matt Sarrasin, Peniel Bustamante Villalobos, B. Franz Lang, Gertraud Burger

**Affiliations:** Département de Biochimie, Robert Cedergren Centre for Bioinformatics and Genomics, Université de Montréal, Montréal H3T 1J4, QC, Canada

**Keywords:** 28S-rRNA phylogeny, biofertilization, *Codinaeella* sp., endophyte, ericoid mycorrhizal fungus (ErMF), *Vaccinium macrocarpon* Aiton (American cranberry)

## Abstract

Ericaceae thrive in poor soil, which we postulate is facilitated by microbes living inside those plants. Here, we investigate the growth stimulation of the American cranberry (*Vaccinium macrocarpon*) by one of its fungal endosymbionts, EC4. We show that the symbiont resides inside the epidermal root cells of the host but extends into the rhizosphere via its hyphae. Morphological classification of this fungus is ambiguous, but phylogenetic inference based on 28S rRNA identifies EC4 as a *Codinaeella* species (Chaetosphaeriaceae, Sordariomycetes, Ascomycetes). We sequenced the genome and transcriptome of EC4, providing the first ‘Omics’ information of a Chaetosphaeriaceae fungus. The 55.3-Mbp nuclear genome contains 17,582 potential protein-coding genes, of which nearly 500 have the capacity to promote plant growth. For comparing gene sets involved in biofertilization, we annotated the published genome assembly of the plant-growth-promoting *Trichoderma hamatum*. The number of proteins involved in phosphate transport and solubilization is similar in the two fungi. In contrast, EC4 has ~50% more genes associated with ammonium, nitrate/nitrite transport, and phytohormone synthesis. The expression of 36 presumed plant-growth-promoting EC4 genes is stimulated when the fungus is in contact with the plant. Thus, Omics and *in-plantae* tests make EC4 a promising candidate for cranberry biofertilization on nutrient-poor soils.

## 1. Introduction

Essentially all plants in natural habitats are associated with microbial symbionts, which colonize the exterior or interior of roots, stems, leaves or seeds [1,2]. A symbiont that can be found inside plant tissues is referred to as an endophyte, wherein it occupies either the space between (intercellular) or within plant cells (intracellular) [3]. Endophytes belong to all domains of life, including Archaea, Bacteria, and Eukarya [4,5]. Among the latter are protists such as Oomycota, Rhizaria and Apicomplexa [6,7], but most belong to Fungi.

In its original definition, ‘endophyte’ designates a microbe that lives within a host organism such as a plant [8]. However, in the literature, the term is often redefined to describe the nature of the interaction between the symbiont and the plant host. For instance, microbes found inside a plant that do not elicit disease symptoms are often referred to as ‘true’ endophytes, while those causing diseases are instead considered pathogens to the exclusion of endophytes [9]. However, a definition based on the effect on the plant host is ambiguous and can be misleading. It is increasingly acknowledged that microbe-plant interactions fall on a spectrum that ranges from pathogenic to neutral to mutualistic relationships [10]. Further, these relationships are dynamic, exemplified by dormant pathogens that are activated under stress conditions [11] or a microorganism that promotes growth in one plant host but triggers disease in another [12,13]. In the following, we will therefore use ‘endophyte’ in its original sense to refer to a microbe found inside plant tissues [10,14] or synonymously with plant endosymbiont. This broad definition implies that a relationship can change with the life cycle, environmental factors, and host taxon of a particular microbe rather than being a feature of that organism itself [15].

Arguably, the most commonly investigated fungal endophytes are the Arbuscular Mycorrhizal Fungi (AMF). The term ‘mycorrhiza’ designates an association between microbes (that live either on the outer surface or inside plant tissues) and plant roots. AMF, which colonize more than 90% of all land plant species [16], are the most widespread fungal root endophytes. Molecular phylogenetic analysis places AMF in a coherent, monophyletic clade, i.e., the Glomeromycota [17].

Among the few plant groups that do not harbor AMF are Ericaceae, a prominent family of flowering plants with more than 100 genera and 4000 species [18]. Ericaceae are associated with fungi called Ericoid Mycorrhizal fungi (ErMF) instead of AMF. However, the definition of ErMF used in the current literature is inconsistent. Some authors use the term for all the endomycorrhizal fungi of Ericaceae [19], whereas others restrict this definition to those that form dense, coiled hyphae of a given shape within the epidermal root cells, and optionally, sheath-like structures in the rhizosphere surrounding the roots of the host [20]. In stark contrast to AMF, ErMF are the most diverse assortment of fungi belonging to the Ascomycota and Basidiomycota [19], suggesting either convergent evolution or horizontal gene transfer in creating the ‘typical’ ericoid mycorrhizal phenotype [21]. Therefore, fungal endophytes described in the literature as ErMF have little in common with each other except that they colonize ericacean plants and are unrelated to AMF.

Studies focusing on the endosymbionts of Ericaceae of the genus *Vaccinium* (cranberry) focus mostly on plant pathogens [22,23,24]. To our knowledge, only two studies reported cranberry endophytes that are possibly saprophytic, or of possibly latent pathogenicity [25,26], and two other publications describe the beneficial effect of cranberry symbionts, including the suppression of a pathogen [27] and the stimulation of nitrogen influx into the plant [28].

A more recent study of our laboratory explored ErMF systematically by surveying the microbiome of *Vaccinium macrocarpon* Aiton (American cranberry) [29]. Nearly 60 different endophytic fungi were isolated and classified by ribotyping as members of at least ten distinct classes of Leotiomyceta (Ascomycota) [21]. *In-plantae* tests showed that certain endosymbiont isolates promote the growth of their host (bio-fertilizers), while others suppress the growth of cranberry plant pathogens (biocontrol agents) or have no notable impact on the plant. One of the isolates, Endophytic Champignon 4 (EC4), is particularly interesting as it strongly stimulates the growth of *V. macrocarpon*. It has been classified as *Codinaea* (Chaetospheriaceae) [30], a group of free-living soil fungi with only five endophytic members currently known [31].

The present study reports the morphological characterization and phylogenetic placement of EC4, and growth tests of EC4-colonized plants. Further, genomic and transcriptomic analyses were undertaken to shed light on the molecular basis of the biofertilization conferred by this fungus.

## 2. Materials and Methods

### 2.1. Culture Condition and Preparation of EC4 Inoculum

EC4 was grown on a standard fungal growth medium of Glycerol Yeast Extract (GYE) (Yeast extract—4 g, Glycerol 40 mL—50% stock, Distilled water 760 mL, pH 7) liquid medium and Glycerol Yeast Extract Agar plates (GYEA). Potato dextrose agar plates were used as a culture medium to isolate fungal endosymbionts from plants. To prepare the EC4 inoculum, the fungus was propagated in a liquid medium, then disrupted in a blender (Hamilton Beach, model 51109C, 225 W for 3 min). The titer of colony-forming units (CFUs) was adjusted to 1 × 10^6^ CFU per mL.

### 2.2. Cranberry Plantlet Growth

Cranberry seeds from the Stevens cultivar were surface-sterilized according to [32], including washes with detergent, sodium hypochlorite and ethanol. After sterilization, seeds were placed on a petri dish containing a standard solid minimum mineral growth medium (MMGM) (Table S1). Seeds were monitored visually for contamination. Once germinated (after about four weeks), seeds were aseptically transferred to a culture box containing MMGM. Plantlets were grown in 16 h light and 8 h dark conditions at room temperature.

### 2.3. In-Plantae Experiments with Seedlings

Cranberry seeds were sterilized and grown aseptically in culture boxes (three seeds per condition and three replicates for each condition). For certain tests, the phosphate source of the standard plant growth medium (KH_2_PO_4_∙3H_2_O; Table S1) was replaced with either tricalcium phosphate (Ca_3_(PO4)_2_), phytate (C_6_H_17_NaO_24_P_6_) or hydroxyapatite (Ca_5_(PO4)_3_(OH)). For each growth condition, nine replicates were produced. Plant samples were collected 30 days after inoculation. The effect of EC4 on plant growth was measured by the weight of roots and shoots after drying these tissues in an oven for 16 h at 70 °C. Statistical analysis was performed with “stats” v3.6.2 from the Analysis of Variance (ANOVA) package available for R.

### 2.4. Microscopy

After 60 days of growth, cranberry plantlets were inoculated with EC4 as follows. A suspension of mechanically disrupted hyphae was prepared at a titer of ~1 × 10^8^/mL CFUs, of which 10 µL (~1 × 10^6^ CFUs) were added to the agar near the plantlet’s roots. Plants were ‘sacrificed’ after four weeks. Cranberry roots, shoots and leaves were stained with Solophenyl Flavine followed by Safranin as described elsewhere [33]. Three plants from three independent culture boxes were examined by light and fluorescence microscopy. For the latter technique, stained samples were mounted in 50% (*v/v*) glycerol and viewed under a Nikon Eclipse Ts2R. Images were processed using the NIS elements online deconvolution test site (https://deconv.laboratory-imaging.com/process, accessed on 22 February 2021).

### 2.5. DNA Isolation from Plant Roots, Polymerase Chain Reaction and Sanger Sequencing

DNA was extracted from plant roots grown in contact with EC4. Roots from a single plant were surface-sterilized and cut into small pieces, DNA was isolated and purified using the Qiagen DNeasy PowerPlant Pro Kit according to the manufacturer’s protocol. PCR was performed with the fungus-specific primers BMBC-F and ITS4-R [34] (Table S2). Amplicons were separated by agarose gel electrophoresis, purified using the QIAquick Gel Extraction Kit, and sequenced by the technology platform at the IRIC, UdeM, using the PCR primers mentioned above.

### 2.6. DNA Isolation, Library Construction, and Whole-Genome Sequencing

GYE liquid medium was inoculated with ~1 × 10^6^ CFUs of an EC4 hyphal suspension. The culture was grown for three days at room temperature in a shaking incubator. Genomic DNA was isolated from a 50-mL liquid culture of EC4 using the DNeasy Plant Mini Kit according to the manufacturer’s recommendation. Both the library preparation and the Illumina sequencing were outsourced to the sequencing technology platform of the Genome Quebec Innovation Center in Montreal. Illumina MiSeq paired-end sequencing yielded a read length of 300 bp.

### 2.7. Growth Conditions, RNA Isolation, RNA-Seq Library Construction, Sequencing, and Differential Gene Expression Analysis

**Figure 1:**
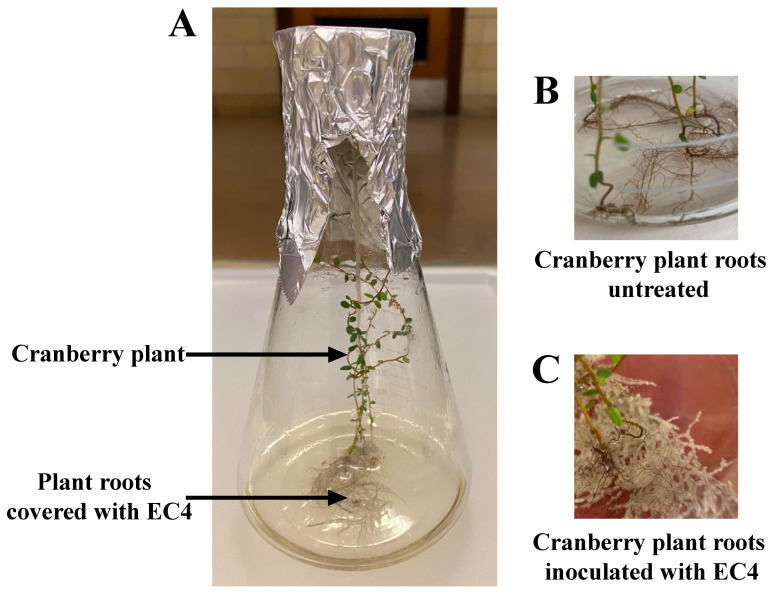
Cranberry plant inoculated with EC4 and cultivated in liquid medium. Microbe-free cranberry plantlets were first grown in a minimum mineral medium for three months, then transferred to a yeast-glycerol medium inoculated with EC4. (**A**) Experimental set-up. (**B**) Roots of control plant in medium without EC4. (**C**) Mycelium-covered plant roots in EC4-inoculated medium.

To test RNA expression of EC4 in contact with its host, ~1 × 10^6^ CFUs of EC4 were inoculated into a liquid 50 mL GYE medium, in which the roots of a six-month-old live cranberry plantlet were suspended (Figure 1). The control experiments were performed without plantlets. Cultures were grown for three days at room temperature in a shaking incubator. The total RNA of EC4 was isolated using the RNeasy Plus Universal kit from Qiagen following the manufacturer’s recommendations. Construction of poly-A RNA stranded libraries and Illumina NovaSeq paired-end sequencing with 100-bp read length were outsourced to the Genome Quebec Innovation Center in Montreal. Reads were trimmed of adapter sequences using Trimmomatic (v0.35) [35] and aligned to the genome using STAR v2.7.1 [36] with default parameters. Differential gene expression analysis was performed using DESeq2 [37] using three biological replicates for each condition. Genes with a log2 fold change of ≥+1 and ≤−1 were considered up-regulated and down-regulated, respectively, using an FDR cutoff of 0.05.

### 2.8. De-Novo Genome Assembly and Structural and Functional Annotation

Illumina MiSeq reads were trimmed of adapter sequences using Trimmomatic (v0.35) [35], and reads were corrected using the k-mer-based error corrector Rcorrector (v1.0.4) [38]. The nuclear genome of EC4 was assembled *de novo* employing the SPAdes assembler (v 3.15.0) [39]. Structural genome annotation was performed with an in-house pipeline [40] that integrates gene-model predictions based on transcriptome data and comparisons with known protein sequences. Product names of protein-coding genes were transferred from the top BLAST hit against the UniProt-reviewed database [41], and domain-specific information was obtained from searches in the PFAM database [42]. The annotation procedure is described in more detail in Method S1. The mitochondrial genome of EC4 was assembled using an in-house script built on the SPAdes assembler and annotated with the MFannot web server (https://megasun.bch.umontreal.ca/apps/mfannot/, accessed on 16 January 2020) (B.F. Lang, unpublished). The nuclear and mitochondrial genome assemblies, annotation files, and RNA-Seq data sets of EC4 were deposited under the NCBI BioProject ID: PRJNA831867. Gene Ontology term assignments were carried out using Blast2GO (v6.0.3) with default settings [43]. The ploidy of EC4 was estimated using GenomeScope v2.0 [44]. Transposable elements in the EC4 genome were identified using RepeatMasker v4.1.3 [45], which uses curated transposable element models in the Dfam database. The assembly of *Trichoderma hamatum* (GCA_000331835.2) was downloaded from the NCBI genome server, and RNA-Seq (SRX996826, SRX996827 and SRX996828) data were obtained via the NCBI SRA database. Structural and functional genome annotation was done the same way as for EC4. Orthologous gene clusters were annotated and compared between EC4 and *Trichoderma hamatum* using OrthoVenn2 [46].

### 2.9. HMM Profile Construction and Search for Homologous Proteins

Protein sequences of plant growth-promoting genes were collected from the UniProt database (SwissProt and TrEMBL) (Table S3) and used to perform a BLAST search against all the fungal sequences available in the NCBI RefSeq database. Sequences matching with an E-value of 1e-7 and lower were used to build multiple sequence alignments with Muscle [47]. Alignment columns with gaps in more than 20% of sequences were discarded via the trimAl program v1.2 [48]. The resulting alignments were used for building profile HMMs employing the HMMER suite v3.3 [49]. The profile HMMs were used to search with hmmsearch (default parameters) from the HMMER suite for homologs in the EC4 proteome. Some proteins had significant hits with several profile HMMs (for example, p450-1, p4502, p450-3, p450-4 and p450/FCK2). In these cases, the HMM producing the lowest e-value was used to assign protein function. Proteins were grouped into families using phmmer [49] of the HMMER suite and Markov clustering (MLC) [50].

### 2.10. Phylogenetic Analysis

The phylogenetic tree was constructed using 28S rRNA sequences (~830 bp length) available from the NCBI RefSeq Targeted Loci Project [PRJNA51803]. The multiple sequence alignment was generated as described above. The tree was inferred with RAxML-HPC v.8.2.12 [51] using GTRCAT approximation and bootstrapping with 1000 replicates and visualization with the iTOL program [52].

## 3. Results and Discussion

### 3.1. EC4 Forms Distinctive Hyphal Structures Outside Plant Roots and Inside Root Cells

**Figure 2:**
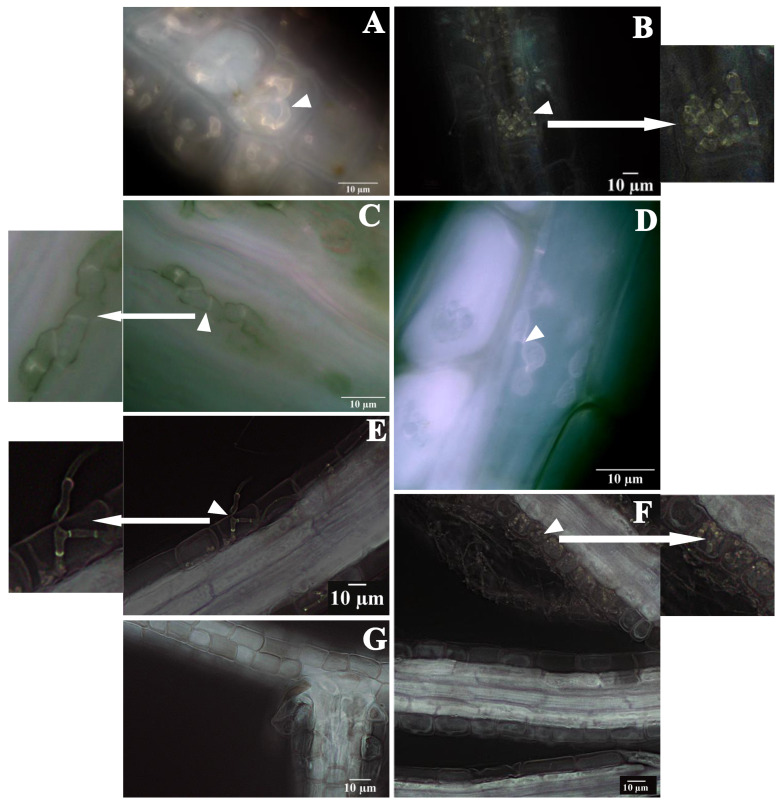
Microscopy of cranberry roots colonized by EC4. Plant tissues and fungal hyphae were differentially stained using solophenyl flavine and Safranin and inspected by light and fluorescent microscopy. (**A**,**B**) Loose hyphal coils of EC4 (arrowheads) inside cranberry root cells. (**C**,**D**) EC4 hyphae with thick, melanized septate hyphae (arrowheads). (**E**), EC4 hyphae (arrowhead) inside an epidermal cell of the host root. (**F**) EC4 hyphae (arrowhead) wrapped around the plant’s roots. (**G**) Roots of control plant not inoculated with EC4.

The fungal endophyte EC4 was initially isolated from the roots of *V. macrocarpon* (cultivar Stevens) [21]. To investigate if EC4 also colonizes other plant tissues, endophyte-free cranberry seedlings were inoculated with the fungus and subsequently examined by light and fluorescence microscopy. Differential staining (see Methods) clearly distinguishes between plant cell walls, fungal cell walls, and fungal septa. Hyphae were not detected in the stem or leave samples, but in the roots, where they form complex structures of loose coils inside epidermal and some cortical cells (Figure 2A,B) and thick, pigmented septate hyphae inside cortical cells (Figure 2C,D). EC4-hyphae are also attached to the surface of the roots (Figure 2E,F). The microscopically detected endophyte was confirmed to be EC4 by two approaches. First, endophytes were isolated by micromanipulation from microscopically examined material and cultivated on agar plates; the growth phenotype of the resulting colonies was the same as that of EC4 (Figure S1). Second, the DNA extracted from such colonies had an identical ribosomal Internal Transcribed Spacer (ITS) sequence as that of EC4 (Result S1).

Rigorous morphology-based taxonomy of fungi relies heavily on the arrangement of conidiophores and conidial shape. However, as earlier described, sporulation of ErMFs (and EC4) could not be triggered. Some authors have classified ErMFs based on the hyphal shape formed inside plant cells. The structures observed in EC4 are reminiscent of the coils defined by others as typical for ErMF [53]. However, the posited characteristics of ErMF are most likely a homoplasmy resulting from the convergent evolution of the ascomycete and basidiomycete taxa currently united in this group. Thus, the classification of EC4 and ErMFs requires molecular phylogeny.

### 3.2. Molecular Phylogeny Places EC4 in the Codinaeella Genus

EC4 was initially classified by ribotyping as a *Codinaea* species (Chaetospheriaceae, Sordariomycetes, Ascomycota) [21], as its ITS sequence was most similar to that of GenBank acc. nr. MN864188.1, which was previously labelled *Codinaea* sp. [14]. We verified this assignment by phylogenetic analysis of all fungal isolates reported in our initial study [21] using ITS sequences. Indeed, the tree groups together coherently Sordariomycetes species, with EC4 and the published *Codinaea* sp. (MN864188.1) forming sister taxa (Figure S2).

A recently published molecular phylogeny based on ITS, 28S, and tef1-α sequences subdivided the traditional *Codinaea* into six genera, the ‘true’ *Codinaea* plus *Codinaeella*, *Stilbochaeta*, *Nimesporella*, *Tainosphaeriella*, and *Xyladelphia* [31]. To pinpoint the genus to which EC4 belongs, we built a phylogeny with 28S rRNA sequences from a set of expert-confirmed Chaetospheriaceae available in the NCBI Targeted Gene Loci collection. This resource adheres to the amended taxonomy of *Codinaea* and *Codinaea*-like organisms mentioned above.

**Figure 3:**
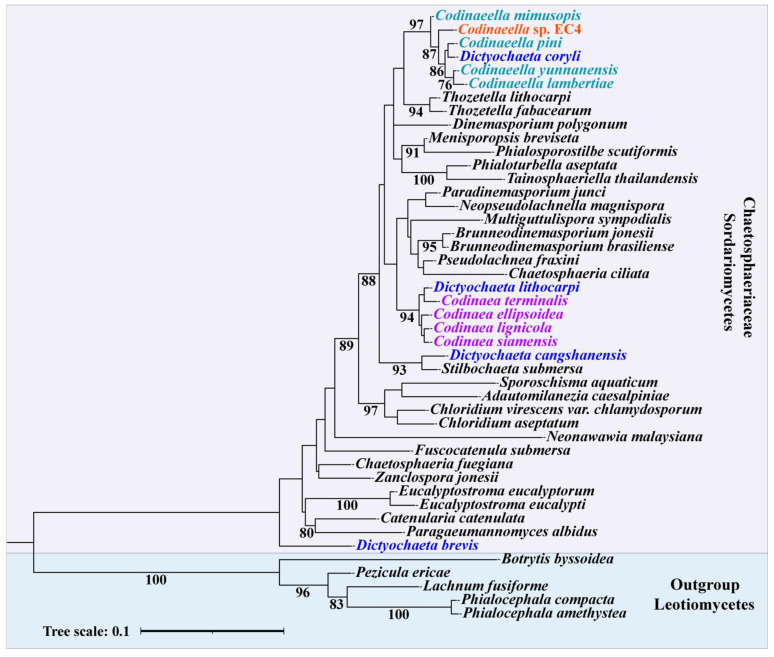
Phylogenetic placement of EC4. The phylogeny is based on 28S rRNA sequences of Chaetospheriaceae and Leotiomycetes (outgroup) available from the NCBI RefSeq Targeted Gene Loci Project [PRJNA51803]. The accession numbers of the species used are listed in Supplementary Table S4. The tree was inferred with RAxML [51] using the GTRCAT model. Only bootstrap support values ≥ 75 are shown. EC4 groups together with the taxa of the *Codinaeella* genus, with a support value of 97%.

The phylogenetic tree (Figure 3 and Figure S3) confirms our initial higher-level classification with Chaetospheriaceae grouping coherently together, supported by strong (100%; e.g., *Dinemasporium*) to moderate bootstrap values (~92%; e.g., *Codinaea*). EC4 is placed with substantial support (97%) within the new genus *Codinaeella*; hence, we consider EC4 a member of this taxon. The tree also shows that among the well-studied ascomycetes, the closest relatives of EC4 are *Neurospora* and *Trichoderma* (Sordariomycetes; Figure S4). Note that, in contrast to other Chaetospheriaceae, *Codinaeella* sp. are rarely reported to be endophytes, with one of the exceptions being *Codinaeella lambertiae* PDD 110188 (MN864188.1) [54].

Finally, we note, as others did before [31], that *Dictyochaeta* species do not form a coherent clade but are scattered across the tree. For instance, *D. coryli* associates with *Codinaeella* (Figure 3 and Figure S3). Indeed, due to considerable morphological similarities, *Dictyochaeta* and *Codinaea* species have changed their taxonomic affiliations several times in the recent past [31].

### 3.3. EC4 Promotes the Growth of Cranberry Plantlets

**Figure 4:**
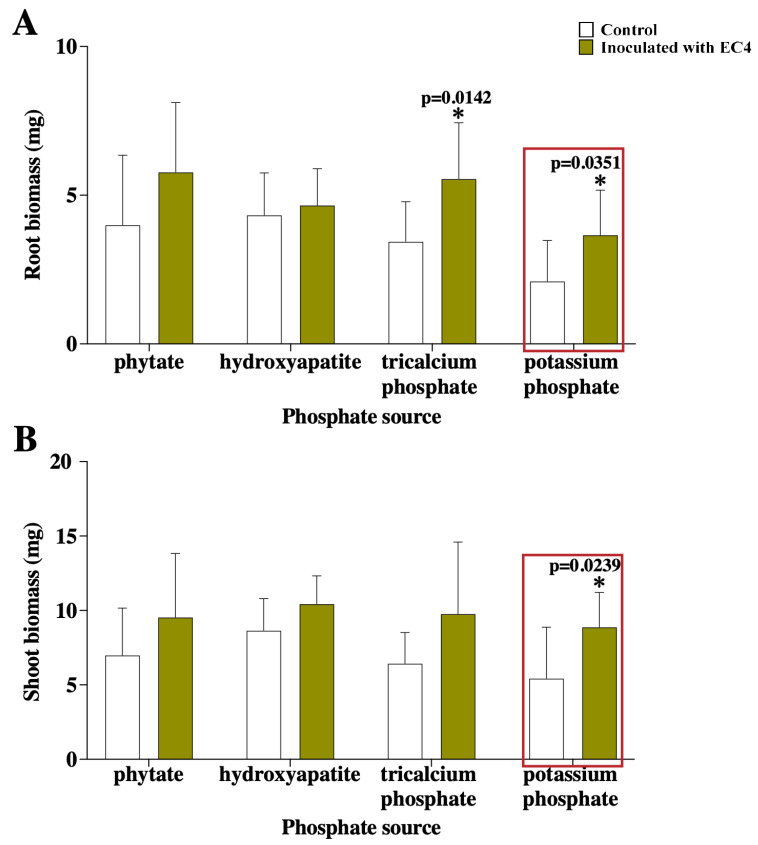
Growth tests of EC4-inoculated cranberry plantlets. Plants were cultivated in the greenhouse on a solid minimum mineral growth medium containing either the standard (soluble) potassium phosphate or insoluble phosphorus sources. The roots and shoots were harvested after 30 days, dried and weighed. Biomass of roots (**A**) and shoots (**B**) from plants grown on a medium containing potassium phosphate or the insoluble phytate, hydroxyapatite or tricalcium phosphate as a phosphorus source. Compared to the controls, EC4 significantly stimulates biomass accumulation in roots from plants grown on potassium phosphate (*p* = 0.0351) and tricalcium phosphate (*p* = 0.0142), as well as in shoots from plants grown on potassium phosphate (*p* = 0.0239). Olive-green-filled bars, plants inoculated with EC4; white-filled bars, controls ‘inoculated’ with water. Whiskers represent the standard deviation, and the star indicates a statistical significance of *p* < 0.05.

Our initial study [21] reported EC4-induced growth stimulation of cranberry plants. More specifically, cuttings were taken from young runners of field-grown plants and surface-disinfected. To exclude a potentially unnoted, pre-existing microbial-endophyte colonization of the cuttings, we repeated the growth-stimulation test with microbe-free plantlets grown from seeds. The results with plantlets confirm the findings obtained with cuttings: after cultivating the plantlets for one month on a standard synthetic medium (containing potassium phosphate (KH_2_PO_4_∙3H_2_O) as the sole phosphorus source), the biomass of roots and shoots is considerably higher in EC4-inoculated plants compared to the controls (Figure 4A,B, boxed).

We also measured the biomass production on media containing phosphorus sources that typically plants assimilate only poorly, notably phytate (inositol polyphosphate, an organic-phosphate storage form synthesized by plants that they cannot take up from the soil), hydroxyapatite, and the water-insoluble tricalcium phosphate (Ca_3_(PO_4_)_2_). On the latter, the root biomass is considerably higher in EC4-inoculated plantlets compared to the controls. In contrast, no difference is detected when hydroxyapatite or phytate is the sole phosphorous source (Figure 4A,B). The latter result seems at odds with the earlier report stating that EC4 solubilizes phytate on agar plates [21]. However, the design of the two experiments is not comparable because the plate test involved many more EC4 cells than the *in-plantae* test described here. The few endophyte cells inside the host presumably solubilized an amount of phytate that was insufficient to stimulate plant growth.

As far as we know, there is only a single report by another group than ours on endophyte-conferred growth stimulation in cranberry. The corresponding study shows that the fungus *Pezizella ericae* increased the influx of radioactively-labelled nitrogen into cranberry [28], which corroborates our genome and transcriptome-based findings described in the following sections. Interestingly, *P. ericae* was initially isolated from another ericaceous plant, the common heather (*Calluna vulgaris*).

### 3.4. The Nuclear and Mitochondrial Genomes of EC4

We sequenced the mitochondrial and nuclear genomes of EC4 with the Illumina technology to investigate the genes of EC4 that may be involved in plant-growth stimulation. The nuclear genome was assembled into 359 contigs with a cumulative length of ~55.3 Mbp (Table 1). A k-mer-based analysis of sequencing reads predicts a diploid genome (Figure S5).

The assembly of chromosome-sized nuclear contigs was hampered by long repetitive DNA regions mostly containing transposable elements (described in detail below). Nonetheless, benchmarking against the universal single-copy orthologous gene complement (BUSCO [55]) using the Sordariomycetes dataset indicates that the EC4 genome assembly contains nearly 99% of the expected orthologs, with only 33 missing. The ploidy level of EC4 is typical for fungi in general, whereas the genome size and gene number of EC4 are somewhat larger compared to the ascomycete average [56,57].

Automated structural annotation of the nuclear EC4 genome by an in-house-developed pipeline (see Methods) predicts about 17,500 protein-coding genes (Table 1). Nearly 89% of the predicted proteins were assigned an informative product name based on the top BLAST hit in SwissProt. Of the rest, ~1% contain a conserved Pfam protein domain, whereas 10% are of unknown function (hypothetical proteins).

Nearly 20% (3,492) of nuclear protein-coding EC4 genes were predicted to produce mRNA isoforms, predominantly by intron retention. The observed percentage appeared to be unusually high, as alternative splicing has been considered to occur only rarely in fungi. To exclude potential errors in gene modelling, 21 randomly chosen genes were visually inspected regarding the coverage of RNA-Seq reads. None of the gene structures were based on incorrectly aligned ‘split’ RNA-seq reads, corroborating frequent alternative splicing in EC4. Recent genomic studies detected a growing number of fungi with alternatively spliced genes. For example, the proportion of such genes is nearly 50% in a *Trichoderma* species, with intron retention being the predominant type of alternative splicing [58].

The mitochondrial DNA of EC4 forms a single circular-mapping contig of nearly 32 kbp with a high A+T content (74.2%). The genome codes for the common 14 proteins involved in electron transport and energy conservation, 23 tRNAs, two ribosomal RNAs and eight Open Reading Frames (ORFs) of unknown function (Table S5). Only two introns were identified. One belongs to group IA and is inserted in the *rnl* gene (specifying the large subunit ribosomal RNA), whereas the other is a group IB intron residing in the *cox1* gene (encoding subunit 1 of cytochrome oxidase). Mitochondrial nucleotide composition, gene complement, and intron types of EC4 are common for fungi.

### 3.5. Repeat Regions in the Nuclear Genome of EC4

Nearly 2% of the EC4 nuclear genome assembly consists of transposable elements, notably 75 DNA transposons and more than 520 retroelements (Table S6). In addition, more than 60 of the 410 contigs contain conspicuously A+T-rich blocks (average A+T content and length 77% and ~7800 bp, respectively). First discovered in *Neurospora crassa*, such regions have been associated with Repeat-Induced Point (RIP) mutations [59]. The RIP pathway is a fungus-specific defence mechanism that mitigates the deleterious consequences of proliferating repeat regions and transposable elements by mutating repeats via cytosine-to-thymine transitions. *In-silico* analyses predict that 4% of the EC4 genome had undergone RIP mutations, pinpointing nearly 200 gene-poor Large RIP Affected Regions (LRARs) (Table 1). As in many other fungal genomes [60], LRARs are located close to transposable elements. In EC4, RIP activity is still ongoing, which is corroborated by the presence of genes that encode homologs of the two canonical methyltransferase enzymes implicated in the RIP pathway, DIM-2 (Defective In Methylation) and RID (RIP Deficient). Further, the required homologs of all the cofactors needed for RIP activity in *N. crassa*, notably DIM-3, -5, -7, -8, -9 and HP-1 [60,61,62], are also encoded by the EC4 genome.

### 3.6. The Nuclear Genome of EC4 Harbours Genes Linked to Plant-Growth Promotion

Symbiotic fungi can stimulate the growth of their plant host in different ways. Strategies include the improvement of mineral nutrition via (i) mineral transport from remote soil locations to the plant, (ii) solubilization of water-insoluble minerals, and (iii) facilitation of mineral uptake into plant cells [63]. The other strategy is the fungal synthesis of phytohormones that are taken up by the plant [64].

To identify genes in the EC4 genome that have the potential to stimulate plant growth by improving mineral nutrition, we constructed 13 profile Hidden Markov Models (HMMs) using available homologs of experimentally validated fungal proteins that have been demonstrated in model systems to play a role in the assimilation, uptake and efflux of nitrogen, phosphorus, and potassium (see Table S3, including references). Note that the currently available information is mostly from non-symbiotic fungi such as *Saccharomyces cerevisiae*, *N. crassa*, *Ustilago maydis*, and *Magnaporthe grisea*.

The search with the above profile HMMs retrieved a total of 193 ‘mineral-nutrition’ proteins in the EC4-inferred proteome (Table 2). Most of the HMMs returned multiple (up to 143) significant hits, which represent families of proteins sharing the same functional domain (Data file S1).

More specifically, the EC4 nuclear genome encodes 79 nitrogen-nutrition related homologs that transport nitrate, nitrite, ammonium, and amino acids across cell membranes. In Glomerales and symbiotic basidiomycetes, these transporters are functionally characterized and reported to augment the nitrogen content of their host plant [65,66,67]. In addition, EC4 possesses 87 genes related to phosphorus nutrition. Among these are acid and alkaline phosphatases secreted by many endophytic fungi. By hydrolyzing water-insoluble organic phosphorous compounds, these enzymes increase the phosphate available to the plants (reviewed in [68]). The presence of inorganic-phosphate-transporter homologs in the EC4 genome is consistent with our *in-plantae* experiments showing that EC4 induces growth promotion of plantlets on water-insoluble Ca_3_(PO4)_2_. As detailed below, most (68%) of these genes are transcribed and thus considered functional. The EC4 genome also contains 27 genes involved in potassium uptake (e.g., TRK (TRansporter of K^+^) and ACU (Alkali Cation Uptake transporters) and efflux transporters (e.g., TOK (Tandem-pore Outward-rectifying K^+^ and ENA (Exit NAtrium)). It has been demonstrated that the overexpression of the TRK and TOK genes in the ectomycorrhizal fungus *Hebeloma* augments the potassium supply of its host, pine [69,70]. In addition, the ACU gene has been reported in *U. maydis* to be involved in high-affinity K+ uptake. ENA, which controls the cell’s potassium efflux has been mostly studied in non-symbiotic fungi such as *S. cerevisiae* but is proposed in plant-fungal symbioses to transfer K+ to the interface between the two partners, where plants would then take up K+ using its own K+ transporters [63].

To identify the EC4 genes potentially involved in the production of phytohormones, profile HMMs were constructed for 14 enzymes participating in the pathways involved in indole acetic acid (IAA), gibberellin, and cytokinin synthesis, again using experimentally validated fungal proteins [71,72,73]. All profiles returned significant hits except the one built from CPS/KS (ent-CoPalyl/ent-Kaurene Synthase) proteins, which are gibberellin-biosynthetic enzymes produced e.g., by *Fusarium* [72,74]. In total, we detected almost 341 proteins in the EC4 nuclear genome assembly with a likely role in phytohormone synthesis (Table 2), including protein families with up to ten members (Data file S1). Particularly abundant in EC4 are proteins belonging to the P450 superfamily. With 220 distinct members, the superfamily in EC4 is considerably larger than reported for most other fungi [75]. A notable exception is the basidiomycete fungus *Postia placenta*, which is equipped with nearly 360 P450 genes [76]. These genes are typically arranged in clusters in the nuclear genome. For example, the ~150 P450 genes of the basidiomycete *Phanerochaete* are organized in only 16 arrays suggesting extensive tandem duplications [77]. In EC4, however, the corresponding genes are rather dispersed warranting an investigation of the mechanism by which this gene family expanded in the lineage leading to EC4. Note that the cytochrome P450 family is not only involved in phytohormone synthesis but also in the biodegradation of natural polymers such as lignin and human-made xenobiotic compounds [78].

### 3.7. EC4 Contains a Similar Set of Potential Plant-Growth-Promoting Genes as Trichoderma Hamatum

Among the fungi for which a nuclear genome assembly is available, *Trichoderma hamatum* is the species related most closely to *Codinaeella* sp. EC4. *Trichoderma* forms ectomycorrhizal associations with various plant species and has biofertilization ability that is employed, for instance, in commercial soybean farming [79]. The isolate *T. hamatum* GD12, the genome of which we examine here, is reported to improve the growth of lettuce [80] and *Arabidopsis thaliana* (thale cress) [79]. The published genome assembly (GCA_000331835) of this *T. hamatum* isolate comprises 745 contigs and is 98% complete, according to our BUSCO benchmarking (Table 1). As the *T. hamatum* assembly is not annotated, we predicted gene models and assigned function in the same fashion as for EC4 so that the two genomes are readily comparable.

With only about 10,400 predicted protein-coding genes in the *T. hamatum* assembly, the overall coding capacity is ~40% less than in EC4 (Table 1). The two fungi share ~7000 orthologous gene clusters (see Methods), while a total of approximately 1900 and 500 clusters are unique to EC4 and *Trichoderma*, respectively (Figure S6). GO enrichment analysis of clusters unique to either of the two fungi shows that the only enriched biological process in *Trichoderma* is “transcription, DNA-templated” (GO:0006351). In contrast, EC4 has 13 GO terms enriched, such as transmembrane transport, oxidoreductase activity, and hydrolase activity, indicating that it is catabolically more active than *Trichoderma* (Table S7). Nonetheless, the two fungal genomes include the same types of predicted biofertilization genes, i.e., genes with the propensity of promoting plant growth by mineral supply and phytohormone production. Interestingly, EC4 has about 50% more genes for nitrogen nutrition and phytohormone production than *Trichoderma* (Table 2 and Data file S1).

### 3.8. EC4 Genes That Change Expression in Contact with the Plant

In most studies examining the nature of symbiont genes, the expression of which is altered by the plant host, microbes are cultivated in the presence of plant extract prior to sequencing their transcriptome [81,82,83]. To simulate the microbe-plant interaction more realistically, we cultivated EC4 in the presence (and absence) of the roots from a live cranberry seedling (for the experimental set-up, see Figure 1).

More than 11,000 of the ~17,500 annotated protein-coding EC4 genes have detectable steady-state transcript levels in at least one of the two tested conditions. Of the transcribed genes, 11.6% were differentially expressed in the presence of the host. Gene-Ontology (GO) enrichment analysis of up-regulated genes indicates that EC4, in the presence of cranberry roots, becomes more active as to metabolism (GO terms such as “amino acid metabolic process”, “carboxylic acid metabolic process”) and transport (“nitrogen compound transport”, “organic substance transport”; Table S8).

**Figure 5:**
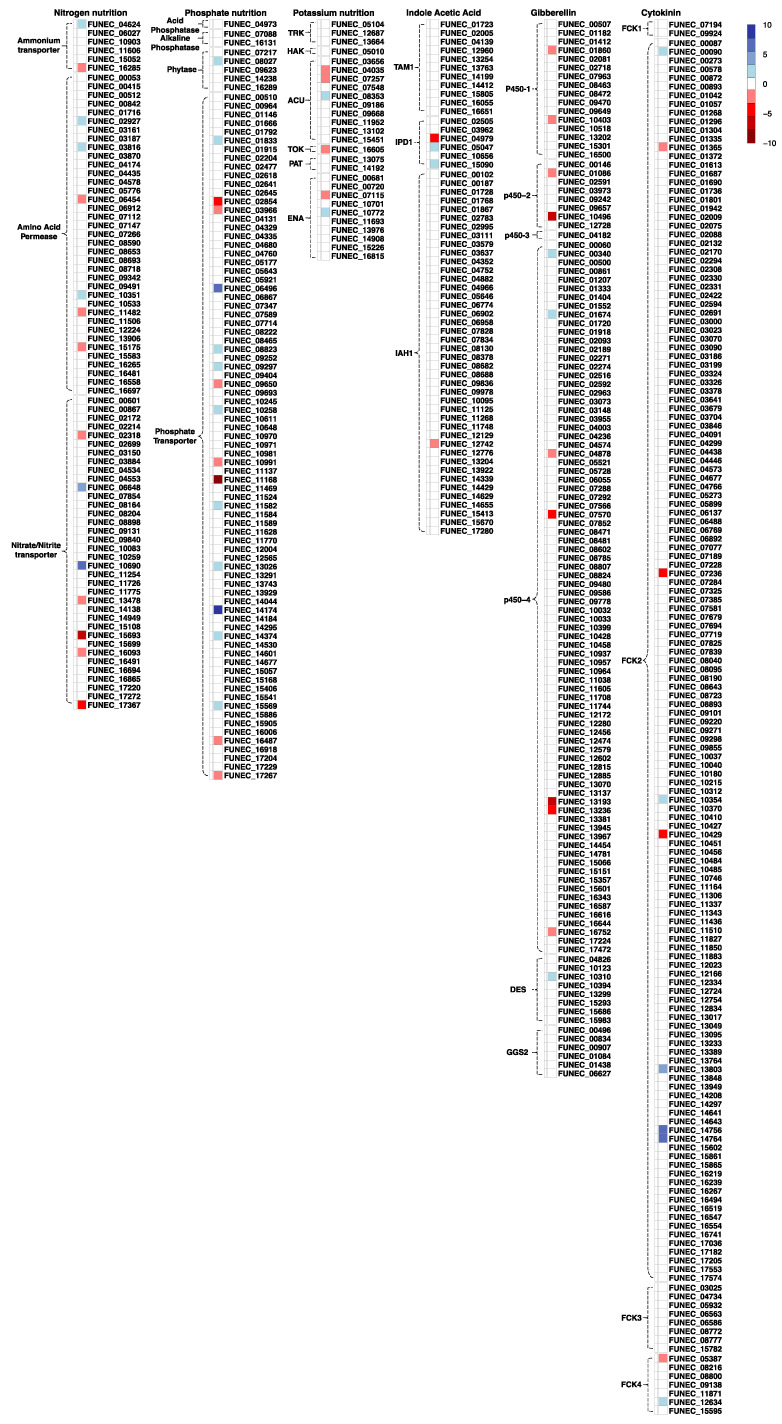
Plant growth-promoting genes of EC4 and their expression when in contact with cranberry plant roots. Top right, color code of the degree of differential expression, with numbers indicating the log2-fold change. Square color-filled boxes show differential expression for each gene. Genes are categorized into classes by dotted parentheses. Abbreviations of genes categories are: ACU—Alkali Cation Uptake transporters, DES—GA4 DESaturase, ENA-Exit Natrium, FCK1—BiFunctional CytoKinin biosynthesis protein, FCK2—cytochrome P450 monooxygenase, FCK3—probable glycosyltransferase, FCK4—probable alcohol acetyltransferase, GGS2—Geranyl Geranyl diphosphate Synthase, HAK—High-Affinity K, IAH1—Isoamyl Acetate-Hydrolyzing esterase 1, IPD1—Indole-3-Pyruvic acid Decarboxylase 1, P450-1—cytochrome P450 monooxygenase 1, P450-2—cytochrome P450 monooxygenase 2, P450-3—cytochrome P450 monooxygenase 3, P450-4—cytochrome P450 monooxygenase 4, PAT—P-type ATPase, TAM1—Tryptophan Aminotransferase 1, TOK—Tandem-pore Outward-rectifying K+, TRK—Transporter of K+.

Of the 193 EC4 genes posited to play a role in nitrogen, phosphorus, and potassium supply to the plant, nearly ¾ are transcribed under one or other examined condition, showing that these genes are indeed functional (Figure 5, Table S9 and Data file S1). Inferred from the presence of introns and codon usage, the remaining genes are most certainly functional as well, but under conditions that we have not tested. About 10% of genes involved in mineral acquisition are only expressed in the presence of cranberry roots (Table S9). Specifically, 12 out of 79 nitrogen-nutrition genes, 11 out of 87 phosphate-nutrition genes, and 3 out of 27 potassium transporters are differentially up-regulated in the presence of cranberry plant roots.

After excluding the multifunctional cytochrome P450 family, about 90 genes remain that are predicted to be specifically involved in the production of phytohormones (gibberellin, indole acetic acid, and cytokine biosynthesis). Of these, at least 53% are expressed in one of the tested conditions. The expression pattern of these genes is quite variable when EC4 is in contact with plant roots. Accurate measurement of change in host-induced phytohormone production would require much larger numbers of replicates and possibly a more stringent control of experimental conditions (Figure 5).

## 4. Conclusions and Outlook

While most published work on cranberry-associated microbes examined pathogens, our research focuses on biofertilizing cranberry symbionts [21]. The fungal symbiont EC4 investigated here is a *Codinaeella* species that significantly stimulates plant growth. Like other mycorrhizal fungi, EC4 forms hyphae that penetrate root cells as well as colonize the root surface. However, an intracellular location is unusual for *Codinaeella* species, which are typically free-living [31]. As of today, no ‘Omics data for *Codinaeella* or any other of the more than 50 chaetosphaeriacean genera is available. Therefore, the EC4 genome reported here represents a valuable reference for future investigations of this large and biologically diverse taxon [31]. In addition, the described results open new research avenues in fungus-plant interactions. For example, it would be interesting to compare the gene complement of the endosymbiotic EC4 isolate with non-symbiotic *Codinaeella* species. Such a comparison promises to shed light on the genetic requirements of endophytic fungi and the origin of the corresponding genes. Further, our transcriptome analyses show that the fungal gene expression pattern is profoundly modulated when the endophyte is in contact with its plant host. Such data are the prerequisite for studying the molecular nature of the communication between EC4 and its cranberry host plant.

From the perspective of research application, cranberry is among the most important ericacean crop plants. Due to its plant-growth-promotion ability, EC4 could become equally impactful for cranberry farming, as is *Trichoderma* for soybean production, by increasing crop yield and reducing the use of chemical fertilizers, which are known to impact human and environmental health [84,85].

## Figures and Tables

**Table 1:** Nuclear genome statistics of EC4 and *Trichoderma hamatum.*

Genome Feature	EC4	*Trichoderma hamatum* ^a^
Nr. of raw reads	10,129,745	/
Raw read length	300 bp	/
Estimated genome coverage	52.5x	/
Size of the nuclear genome assembly	55.3 Mbp	38 Mbp
Nr. of contigs (>200 bp)	359	745
N50	357 Kbp	/
Size of largest contig	1.4 Mbp	/
GC content	52.3%	48.5%
Genome completeness (BUSCO)	98.9% ^b^	98%
Nr. of protein-coding genes	17,582	10,359
Mean gene length	2118 bp	/
Mean exon length	750 bp	/
Nr. of introns	56,315	/
Mean intron length	79 bp	/
Nr. of tRNA genes	225	/
mRNAs with UniProtKB ID	22,385	/
mRNAs with EC numbers	10,699	/
RIP affected genome portion	4.0%	/
Count of LRARs ^c^	196	/
Average size of LRARs	7782 bp	/
Cumulative size of LRARs	1,525,455 bp	/

^a^ A well-studied plant growth promoting fungus; /, information not available; ^b^ Single copy: 98.1%; Duplicated: 0.8%; Fragmented: 0.2%; Missing: 0.9%; n: 3817, ^c^ LRARs, Large RIP Affected Regions.

**Table 2:** Gene families identified in EC4 and *Trichoderma hamatum* with the potential to promote plant growth.

Plant-Growth Promoting Genes ^a^	EC4	*Trichoderma hamatum*
**Nitrogen nutrition**		
Ammonium transporter	6	2
Amino-acid-permease	37	16
Nitrate/nitrite transporter	36	14
**Phosphate nutrition**		
Acid phosphatase	1	1
Alkaline phosphatase	2	1
Phytase	5	3
Phosphate transporter	79	53
**Potassium nutrition—Potassium uptake system**		
TRK (Transporter of K+)	3	1
HAK (High-Affinity K)	1	1
ACU (Alkali Cation Uptake transporters)	10	11
PAT (P-type ATPase)	1	0
**Potassium nutrition—Potassium efflux systems**		
TOK (Tandem-pore Outward-rectifying K+)	2	2
ENA (Exit Natrium)	10	13
**Indole Acetic Acid—biosynthetic genes**		
TAM1 (Tryptophan Aminotransferase 1)	11	4
IPD1 (Indole-3-Pyruvic acid Decarboxylase 1)	6	8
IAH1(Isoamyl Acetate-Hydrolyzing esterase 1)	42	26
**Gibberellin biosynthesis genes**		
CPS/KS (ent-CoPalyl/ent-Kaurene Synthase)	0	0
P450-1 (cytochrome P450 monooxygenase 1) ^b^	16	3
P450-2 (cytochrome P450 monooxygenase 2) ^b^	8	1
P450-3 (cytochrome P450 monooxygenase 3) ^b^	1	0
P450-4 (cytochrome P450 monooxygenase 4) ^b^	82	24
DES (GA4 DESaturase)	8	6
GGS2 (Geranyl Geranyl diphosphate Synthase)	6	3
**Cytokinin biosynthetic genes**		
FCK1 (BiFunctional CytoKinin biosynthesis protein)	2	2
FCK2 (cytochrome P450 monooxygenase) ^b^	143	41
FCK3 (probable glycosyltransferase)	8	4
FCK4 (probable alcohol acetyltransferase)	7	4

^a^ See references in Table S3; ^b^ Enzymes that also function in pathways other than phytohormone synthesis.

## Data Availability

Not applicable.

## Supplementary Materials

The following supporting information can be downloaded at: https://www.mdpi.com/article/10.3390/jof9010126/s1, [86,87,88,89,90,91,92,93,94,95,96,97,98,99,100,101,102,103,104,105,106,107,108] Table S1. Composition of the minimum mineral growth medium for plants; Table S2. Primers used for PCR-amplification of the nuclear ribosomal ITS; Table S3. Experimentally confirmed fungal genes promoting plant growth; Table S4. Data used in the phylogenetic analysis; Table S5. Mitochondrial gene repertoire of EC4; Table S6. Transposable elements in the EC4 genome identified using RepeatMasker; Table S7. GO enrichment analysis^a^ of EC4-specific and *Trichoderma*-specific genes; Table S8. GO enrichment analysis^a^ of differentially up-regulated genes of EC4 when in contact with cranberry plant roots; Table S9. Plant growth-promoting genes of EC4 and their expression; Figure S1. Endophyte re-isolated from EC4-infected cranberry roots; Figure S2. Phylogenetic positioning of fungal cranberry-endophytes reported earlier [21]; Figure S3. Phylogenetic placement of EC4; Figure S4. Schematic phylogenetic tree including EC4 and other well-studied fungi; Figure S5. Ploidy inference of the EC4 nuclear genome; Figure S6. Analysis of orthologous genes in EC4 and *Trichoderma hamatum*; Method S1. Annotation of the EC4 nuclear genome; Result S1. Nuclear ribosomal internal transcribed spacer (ITS) sequence of endophyte re-isolated from EC4-infected cranberry plantlets. Data file S1. Plant growth promoting genes and phytohormones in EC4 and *Trichoderma* and their expression.
