# Supplementary material for: Nuclear Genome Sequence and Gene Expression of an Intracellular Fungal Endophyte Stimulating the Growth of Cranberry Plants
Source: J Fungi (Basel). 2023 Jan 16;9(1):126. doi: 10.3390/jof9010126 (PMC9861600; doi:10.3390/jof9010126)
Supplement: Supplementary file 1 [file jof-09-00126-s001.zip › jof-2104949 Supplementary New.pdf]

Table S1. Composition of the minimum mineral growth medium for plants

Media composition	Concentration
$MgSO_4 \cdot 7H_2O$	730 mg/L
KNO_3	80 mg/L
KCl	65 mg/L
$KH_2PO_4 \cdot 3H_2O$	4.8 mg/L
Ca(NO3)2·4H2O	288 mg/L
KI	0.75 mg/L
NaFe(III) EDTA sodium salt	8 mg/L
$MnSO_4 \cdot H_2O$	4.66 mg/L
$ZnSO_4 \cdot 7H_2O$	2.65 mg/L
H_3BO_3	1.5 mg/L
$CuSO_4$·5H2O	0.13 mg/L
$Na_2MoO_4 \cdot 2H_2O$	2.4 ug/L
Sucrose	100 mg/L
Gelzan	4 g/L
Gamborg's Vitamin Solution [86]	1X

Table S2. Primers used for PCR-amplification of the nuclear ribosomal ITS

Primers	Sequence
BMBC-F	5'-GTACACACCGCCCGTCG-3'
ITS4-R	5′-TTCCWCCGCTTATTGATATGC-3′

Table S3. Experimentally confirmed fungal genes promoting plant growth

Gene function	Fungal species	Accession	References
**Nitrogen nutrition**			
Ammonium transporter	*Rhizophagus irregularis*	XP_025183237	[66]
Amino-acid-permease	*Glomus mosseae*	AAX81451	[87]
Nitrate/nitrite transporter	*Rhizophagus irregularis*	EXX76102	[67]
**Phosphate nutrition**			
Acid phosphatase	*Rhizophagus irregularis*	EXX57538	[88]
Alkaline phosphatase	*Glomus mosseae*	AGC74338	[87]
Phytase	*Aspergillus niger*	P34752	[89]
Phosphate transporter	*Rhizophagus intraradices*	AAL37552	[90]
**Potassium nutrition**[a]			
**Potassium uptake system**			
TRK (Transporter of K^+)	*Neurospora crassa*	XP_957340	[69]
HAK (High-Affinity K)	*Neurospora crassa*	XP_964946	[91]
ACU (Alkali Cation Uptake transporters)	*Ustilago maydis*	XP_011392714	
PAT (P-type ATPase)	*Blastocladiella emersonii*	CAA04499	[92]
**Potassium efflux systems**			
TOK (Tandem-pore Outward-rectifying $K^{+)}$	*Saccharomyces cerevisiae*	NP_012442	[93]
ENA (Exit Natrium)	*Saccharomyces cerevisiae*	NP_010325	[94]
**Indole Acetic Acid – biosynthetic genes**			
TAM1 (Tryptophan AMinotransferase 1)	*Tricholoma vaccinum*	AJP77090	[71]
IPD1 (Indole-3-Pyruvic acid Decarboxylase 1)	*Tricholoma vaccinum*	AJP77091	[71]
IAH1(Isoamyl Acetate-Hydrolyzing esterase 1)	*Tricholoma vaccinum*	AJP77092	[71]
**Gibberellin biosynthetic genes**			
CPS/KS (ent-copalyl/ ent-kaurene synthase)	*Fusarium fujikuroi*	XP_023431478	[72]
P450-1 (cytochrome P450 monooxygenase 1)	*Fusarium fujikuroi*	QGI65211	[72]
P450-2 (cytochrome P450 monooxygenase 2)	*Fusarium fujikuroi*	XP_023431253	[72]
P450-3 (cytochrome P450 monooxygenase 3)	*Fusarium fujikuroi*	XP_023431251	[72]
P450-4 (cytochrome P450 monooxygenase 4)	*Fusarium fujikuroi*	O94142	[72]
DES (GA4 desaturase)	*Fusarium fujikuroi*	XP_023431254	[72]
GGS2 (Geranyl Geranyl diphosphate Synthase)	*Fusarium fujikuroi*	XP_023431252	[72]
**Cytokinin biosynthetic genes**			
FCK1 (Bifunctional CytoKinin biosynthesis protein)	*Fusarium pseudograminearum*	XP_009257765	[73]
FCK2 (cytochrome P450 monooxygenase)	*Fusarium pseudograminearum*	P0DPA4	[73]
FCK3 (probable glycosyltransferase)	*Fusarium pseudograminearum*	XP_009257764	[73]
FCK4 (probable alcohol acetyltransferase)	*Fusarium pseudograminearum*	P0DPA5	[73]

[a]Potassium nutrition genes information available from non-symbiotic fungi and proposed to have a role in plant growth promotion.

Table S4. Data used in the phylogenetic analysis

Accession number	Strain information - 28S rRNA gene sequence from TYPE material
NG_079565.1	*Tainosphaeriella thailandensis* MFLUCC 18-1282
NG_079564.1	*Tainosphaeriella aquatica* MFLUCC 17-2370
NG_067903.1	*Codinaea terminalis* MFLU 19-0214
NG_067563.1	*Stilbochaeta aquatica* MFLU 15-2691
NG_067902.1	*Phialoturbella aseptata* MFLU 19-0208
NG_075392.1	*Stanjehughesia kaohsiungensis* BCRC FU31337
NG_058756.1	*Paragaeumannomyces albidus* PDD 92537
NG_070470.1	*Menisporopsis dushanensis* MFLU 19-0213
NG_070469.1	*Menisporopsis breviseta* MFLU 19-0212
NG_068569.1	*Phialosporostilbe scutiformis* MFLUCC 17-0227
NG_068242.1	*Brunneodinemasporium jonesii* GZCC 16-0050
NG_067904.1	*Dictyochaeta brevis* MFLU 19-0216
NG_067899.1	*Multiguttulispora sympodialis* MFLU 19-0218
NG_067562.1	*Chloridium aseptatum* MFLU 11-1051
NG_059700.1	*Sporoschisma longicatenatum* MFLU 16-1325
NG_058757.1	*Chaetosphaeria metallicans* PDD 92539
NG_081350.1	*Paradinemasporium junci* CBS 148317
NG_081301.1	*Codinaeella mimusopis*
NG_068635.1	*Stilbochaeta submersa* MFLU 18-2321
NG_068634.1	*Codinaea lignicola* MFLU 18-1613
NG_068633.1	*Codinaea ellipsoidea* MFLU 18-1612
NG_059142.1	*Codinaea siamensis* MFLU 15-1149
NG_059053.1	*Codinaeella lambertiae*
NG_058902.1	*Codinaeella pini*
NG_068638.1	*Catenularia catenulata* MFLU 18-1620
NG_068637.1	*Fuscocatenula submersa* MFLUCC 18-1342
NG_067549.1	*Zanclospora jonesii* MFLUCC 15-1015
NG_073871.1	*Chloridium pini* CPC 36627
NG_073859.1	*Dictyochaeta coryli* MFLU 19-1387
NG_073858.1	*Dictyochaeta lithocarpi* MFLUCC 17-2228
NG_073788.1	*Chloridium submersum* MFLUCC 16-1344
NG_073651.1	*Sporoschisma chiangraiense* MFLUCC 18-0703
NG_059017.1	*Paragaeumannomyces garethjonesii* MFLUCC 15-1012
NG_068842.1	*Thozetella pandanicola* MFLUCC 16-0253
NG_068832.1	*Polynema podocarpi* CPC 32761
NG_068777.1	*Sporoschisma aquaticum* DLU 628
NG_068639.1	*Chaetosphaeria aquatica* MFLUCC 18-1341
NG_068636.1	*Dictyochaeta cangshanensis* MFLUCC 17-2214
NG_068632.1	*Tainosphaeria obclavata* MFLUCC 18-0260
NG_068631.1	*Tainosphaeria lunata* MFLUCC 18-0642
NG_068630.1	*Codinaea yunnanensis* MFLUCC 17-0468
NG_068512.1	*Neonawawia malaysiana*
NG_067896.1	*Thozetella neonivea* CBS 145534
NG_067834.1	*Thozetella lithocarpi* MFLU 16-1068
NG_067452.1	*Chaetosphaeria ciliata* CBS 122131
NG_067351.1	*Chloridium vires*cens var. chlamydosporum CBS 114.41

NG_058594.1	*Adautomilanezia caesalpiniae* HUEFS 216632
NG_066268.1	*Chaetosphaeria fuegiana* CBS 114553
NG_064543.1	*Eucalyptostroma eucalyptorum* CPC 31800
NG_059807.1	*Pseudolachnella brevifusiformis* HHUF 30495
NG_059767.1	*Thozetella fabacearum* MFLU 16-1021
NG_059712.1	*Tainosphaeria siamensis* MFLU 15-1142
NG_059410.1	*Pseudolachnella longiciliata* HHUF 27528
NG_059409.1	*Pseudolachnella complanata* HHUF 28282
NG_059408.1	*Pseudolachnella brevicoronata* HHUF 30119
NG_059407.1	*Pseudodinemasporium fabiforme* HHUF 29716
NG_059406.1	*Neopseudolachnella uniseptata* HHUF 29728
NG_059405.1	*Neopseudolachnella magnispora* HHUF 29977
NG_059404.1	*Neopseudolachnella acutispora* HHUF 29727
NG_059257.1	*Eucalyptostroma eucalypti* CPC 28764
NG_059124.1	*Dinemasporium ipomoeae*
NG_059119.1	*Dinemasporium trichophoricola* CBS 136772
NG_059110.1	*Dinemasporium morbidum* CBS 129.66
NG_059109.1	*Dinemasporium polygonum* CBS 516.95
NG_058655.1	*Brunneodinemasporium brasiliense* CBS 112007
NG_057956.1	*Pseudolachnea fraxini*
NG_067901.1	*Kionochaeta castaneae* MFLU 19-0204
NG_067900.1	*Kionochaeta microspora* MFLU 19-0206
NG_069610.1	*Botrytis byssoidea* CBS 104.23
NG_075332.1	*Phialocephala compacta* CBS 507.94
NG_067780.1	*Lachnellula hyalina* CBS 185.66
NG_066455.1	*Lachnum fusiforme* MFLU 15-0230
NG_073819.1	*Phialocephala amethystea* DAOMC 251552
NG_067298.1	*Pezicula microspora* CBS 124641
NG_069888.1	*Pezicula ericae* CBS 120290
NG_068558.1	*Hyaloscypha melinii* CBS 143705
NG_069568.1	*Hyaloscypha finlandica* CBS 444.86
NG_067342.1	*Botrytis fabae* CBS 120.29

Table S5. Mitochondrial gene repertoire of EC4

Gene products	Genes
ATP synthase subunits	*atp6, atp8, atp9*
Cytochrome c oxidase subunits	*cox1, cox2, cox3*
Cytochrome b subunits	*cob*
NADH dehydrogenase subunits	*nad1, nad2, nad3, nad4, nad4L, nad5, nad6*
Open Reading Frames (ORFs) of unknown function	*orf104, orf122, orf219, orf266, orf330, orf356, orf404, orf478*
RNase P	*rnpB*
Large subunit rRNA	*rnl*
Small subunit rRNA	*rns*
tRNA	*trnA(ugc), trnC(gca), trnD(guc), trnE(uuc), trnF(gaa), trnG(ucc), trnH(gug), trnI(gau), trnK(uuu), trnL(uaa), trnL(uag), trnM(cau), trnN(guu), trnP(ugg), trnQ(uug), trnR(acg), trnR(ucu), trnS(gcu), trnS(uga), trnT(ugu), trnV(uac), trnW(uca), trnY(gua)*

Table S6. Transposable elements in the EC4 genome identified using RepeatMasker

	Number of elements[a]	Length occupied	Percentage of sequence[b]
**Retroelements**	521	1,002,462 bp	1.81%
SINEs:	0	0 bp	0.00%
Penelope	0	0 bp	0.00%
LINEs:	112	330,859 bp	0.60%
CRE/SLACS	0	0 bp	0.00%
L2/CR1/Rex	0	0 bp	0.00%
R1/LOA/Jockey	0	0 bp	0.00%
R2/R4/NeSL	0	0 bp	0.00%
RTE/Bov-B	0	0 bp	0.00%
L1/CIN4	0	0 bp	0.00%
LTR elements:	409	671,603 bp	1.21%
BEL/Pao	0	0 bp	0.00%
Ty1/Copia	141	126,375 bp	0.23%
Gypsy/DIRS1	268	545,228 bp	0.99%
Retroviral	0	0 bp	0.00%
**DNA transposons**	75	14,437 bp	0.03%
hobo-Activator	29	5,417 bp	0.01%
Tc1-IS630-Pogo	24	4,478 bp	0.01%
En-Spm	0	0 bp	0.00%
MuDR-IS905	22	4,542 bp	0.01%
PiggyBac	0	0 bp	0.00%
Tourist/Harbinger	0	0 bp	0.00%
Other (Mirage, P, etc.)	0	0 bp	0.00%
**Rolling-circles**	0	0 bp	0.00%
**Unclassified:**	1,967	878,231 bp	1.59%
**Total interspersed repeats:**		1,895,042 bp	3.43%
**Small RNA:**	0	0 bp	0.00%
**Satellites:**	0	0 bp	0.00%
**Simple repeats:**	6,655	271,224 bp	0.49%
**Low complexity:**	656	31,330 bp	0.06%

[a]Repeats fragmented by insertions or deletions have been counted as one element. [b]Proportion of genome

Table S7. GO enrichment analysis[a] of EC4-specific and *Trichoderma*-specific genes

GO ID	Count of *Trichoderma*-specific genes	Count of EC4-specific genes	Name	GO category
GO:0055085	0	60	Transmembrane transport	Biological Process
GO:0006351	27	52	Transcription, DNA-templated	Biological Process
GO:0016491	0	41	Oxidoreductase activity	Molecular Function
GO:0016705	0	26	Oxidoreductase activity, acting on paired donors, with incorporation or reduction of molecular oxygen	Molecular Function
GO:0009405	0	24	Pathogenesis	Biological Process
GO:0016787	0	15	Hydrolase activity	Molecular Function
GO:0004497	0	13	Monooxygenase activity	Molecular Function
GO:0045493	0	13	Xylan catabolic process	Biological Process
GO:0030245	0	13	Cellulose catabolic process	Biological Process
GO:0008643	0	9	Carbohydrate transport	Biological Process
GO:0035442	0	8	Dipeptide transmembrane transport	Biological Process
GO:0015031	0	5	Protein transport	Biological Process
GO:0006412	0	2	Translation	Biological Process

[a] GO enrichment analysis was performed using orthovenn2. See Methods.

Table S8. GO enrichment analysis[a] of differentially up-regulated genes of EC4 when in contact with cranberry plant roots

GO ID	GO Name
GO:0006520	amino acid metabolic process
GO:0019752	carboxylic acid metabolic process
GO:0051641	cellular localization
GO:0070727	cellular macromolecule localization
GO:0051649	establishment of localization in the cell
GO:0045184	establishment of protein localization
GO:0006886	intracellular protein transport
GO:0046907	intracellular transport
GO:0033036	macromolecule localization
GO:0140053	mitochondrial gene expression
GO:0007005	mitochondrion organization
GO:0043386	mycotoxin biosynthetic process
GO:0043385	mycotoxin metabolic process
GO:0071705	nitrogen compound transport
GO:0006082	organic acid metabolic process
GO:0071702	organic substance transport
GO:1901564	organonitrogen compound metabolic process
GO:0043436	oxoacid metabolic process
GO:0006457	protein folding
GO:0008104	protein localization
GO:0015031	protein transport
GO:0019748	secondary metabolic process
GO:0044550	secondary metabolite biosynthetic process
GO:0009403	toxin biosynthetic process
GO:0009404	toxin metabolic process

[a]GO enrichment analysis was performed using the Fisher Exact Test with FDR 0.05. See Methods.

Table S9. Plant growth-promoting genes of EC4 and their expression

Gene class	Nr. of genes					
	**Total**	**Expressed[a]**		**Expressed in contact with plant roots[b]**		
		**Yeast-Glycerol medium**	**Plant Roots**	**Up**	**Down**	**Uncha-nged[c]**
**Nitrogen nutrition (79 genes)**						
Ammonium transporter	6	3	3	1	1	1
Amino-acid-permease	37	16	17	3	3	11
Nitrate/nitrite transporter	36	17	15	2	5	9
**Phosphate nutrition (87 genes)**						
Acid phosphatase	1	1	1	0	0	1
Alkaline phosphatase	2	2	2	0	0	2
Phytase	5	3	3	1	0	2
Phosphate transporter	79	52	43	10	7	29
**Potassium nutrition (27 genes)**						
Potassium uptake system						
TRK (Transporter of K+)	3	2	2	0	0	2
HAK (High-Affinity K)	1	1	1	0	0	1
ACU (Alkali Cation Uptake transporters)	10	8	8	1	2	5
PAT (P-type ATPase)	1	1	1	0	1	0
Potassium efflux systems						
TOK (Tandem-pore Outward-rectifying K+)	2	2	2	0	0	2
ENA (Exit Natrium)	10	9	9	1	1	7
**Indole Acetic Acid - biosynthetic genes (59 genes)**						
TAM1 (Tryptophan AMinotransferase 1)	11	6	6	0	0	6
IPD1 (Indole-3-Pyruvic acid Decarboxylase 1)	6	4	3	2	1	1
IAH1(Isoamyl Acetate-Hydrolyzing esterase 1)	42	19	18	0	1	17
**Gibberellin biosynthetic genes (121 genes)**						
P450-1 (cytochrome P450 monooxygenase 1)	16	7	5	0	2	4
P450-2 (cytochrome P450 monooxygenase 2)	8	2	1	0	2	0
P450-3 (cytochrome P450 monooxygenase 3)	1	0	0	0	0	0
P450-4 (cytochrome P450 monooxygenase 4)	82	33	25	2	5	22
DES (GA4 desaturase)	8	6	5	1	0	4
GGS2 (Geranyl Geranyl diphosphate Synthase)	6	3	3	0	0	3
**Cytokinin biosynthetic genes (160 genes)**						
FCK1 (bifunctional CytoKinin biosynthesis protein)	2	1	1	0	0	1
FCK2 (cytochrome P450 monooxygenase)	143	50	44	5	3	38
FCK3 (probable glycosyltransferase)	8	0	3	0	0	3
FCK4 (probable alcohol acetyltransferase)	7	3	0	1	1	0

[a]Cultured in liquid Yeast-Glycerol medium (control) and in contact with plant roots (test). Genes with TPM values of more than two were considered expressed. [b] Differentially expressed with Fold Change (FC) ≥ +/- 1 and P<0.05. [c]Genes that are expressed in contact with plant roots (4th column) but not differentially expressed were considered unchanged.

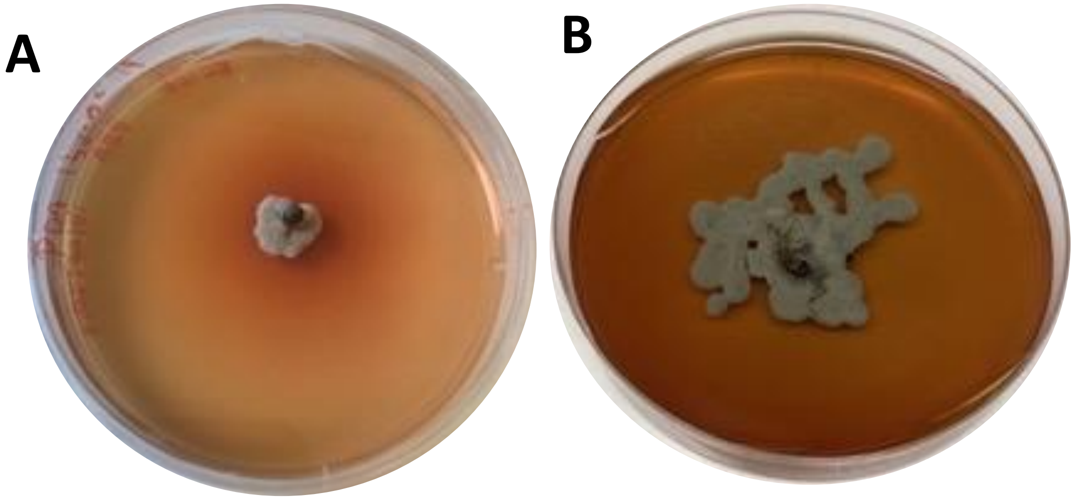

Figure S1. Endophyte re-isolated from EC4-infected cranberry roots. Colony morphology on potato dextrose agar. (A), EC4; (B) endophyte isolated from the roots of a cranberry plant inoculated with EC4. Both colonies have the same morphological features, only that on (B) is larger, and the agar has a darker coloration because of the longer growth time.

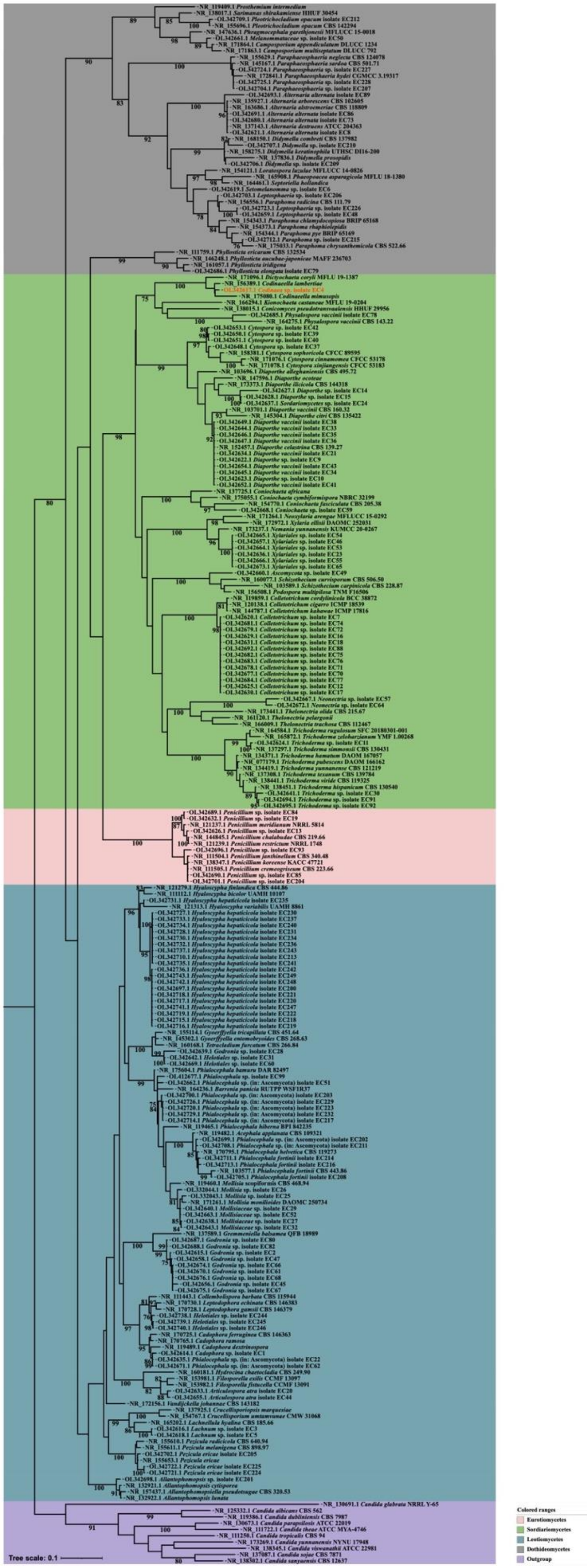
Colored ranges
Eurotiomycetes
Sordariomycetes
Leotiomycetes
Dothideomycetes
Outgroup
Tree scale: 0.1

Figure S2. Phylogenetic positioning of fungal cranberry-endophytes reported earlier [21]. All isolates (labeled with accession number and isolate name) belong to the phylum Ascomycota. Specifically, Leotiomycetes (64 isolates), Sordariomycetes (46 isolates), Dothideomycetes (17 isolates) and Eurotiomycetes (six isolates) class. EC4 groups together with taxa of the Codinaeella genus within the Sordariomycetes class. The other fungal ITS sequences were downloaded from the NCBI nucleotide database. The top three closely related sequences were collected from NCBI RefSeq Targeted Loci Project [PRJNA51803] for each unique fungal endophyte. The tree was constructed with RAxML-HPC v.8.2.12 [51] using the GTRCAT approximation. Bootstrap support values ≥75 are shown. Candida species were used as an outgroup.

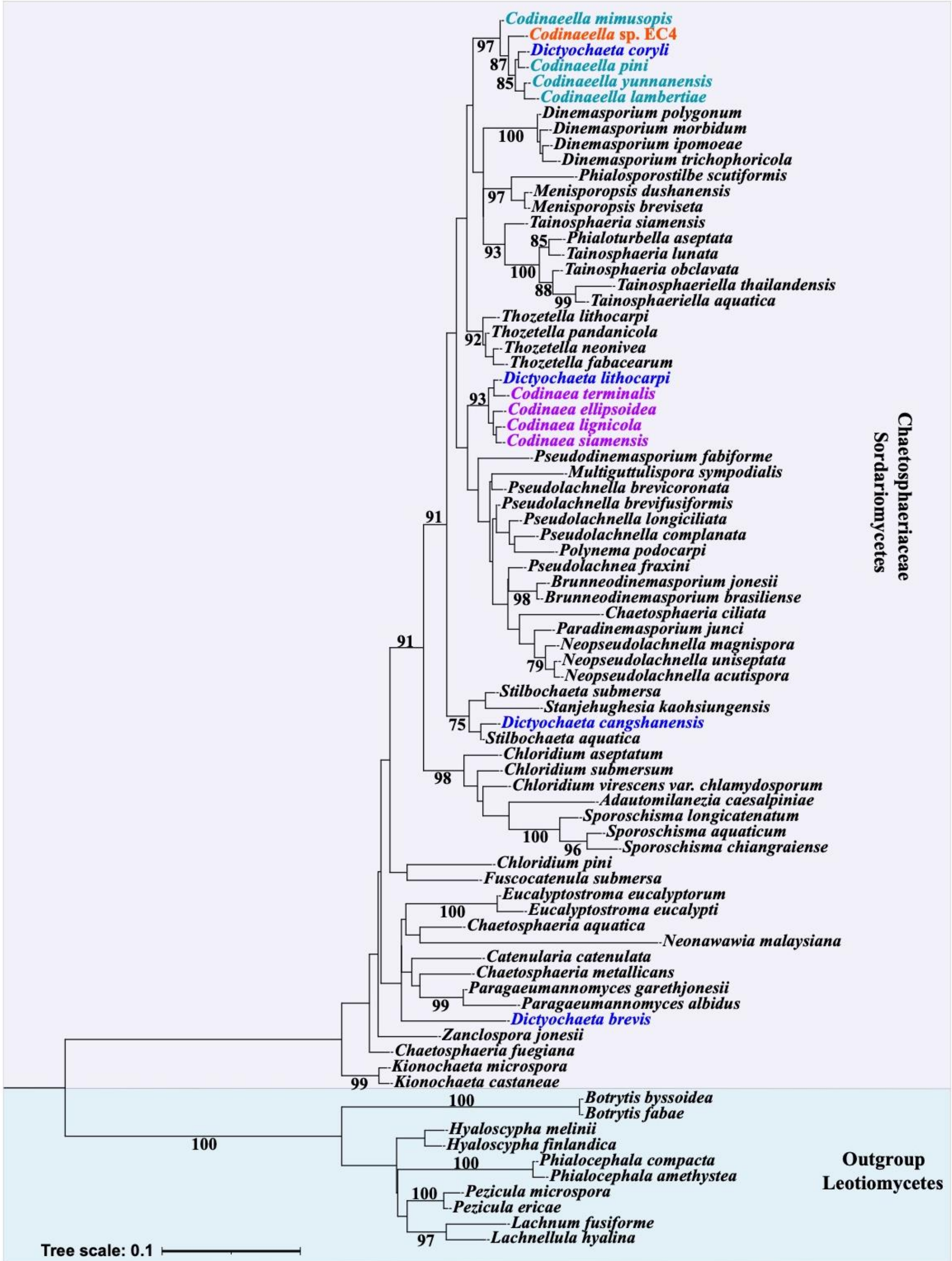

Figure S3. Phylogenetic placement of EC4. Phylogenetic tree based on 28S rRNA sequences from Chaetospheriaceae (class Sordariomycetes) that were downloaded from the NCBI RefSeq Targeted Loci Project [PRJNA51803]. The tree was constructed with RAxML-HPC v.8.2.12 [51] using the GTRCAT approximation. The accession numbers of the species are listed in Supplementary Table S5. Bootstrap support values ≥75 are shown. Leotiomycetes species were used as an outgroup. EC4 groups together with *Codinaeella* species, forming a clade with 97% support.

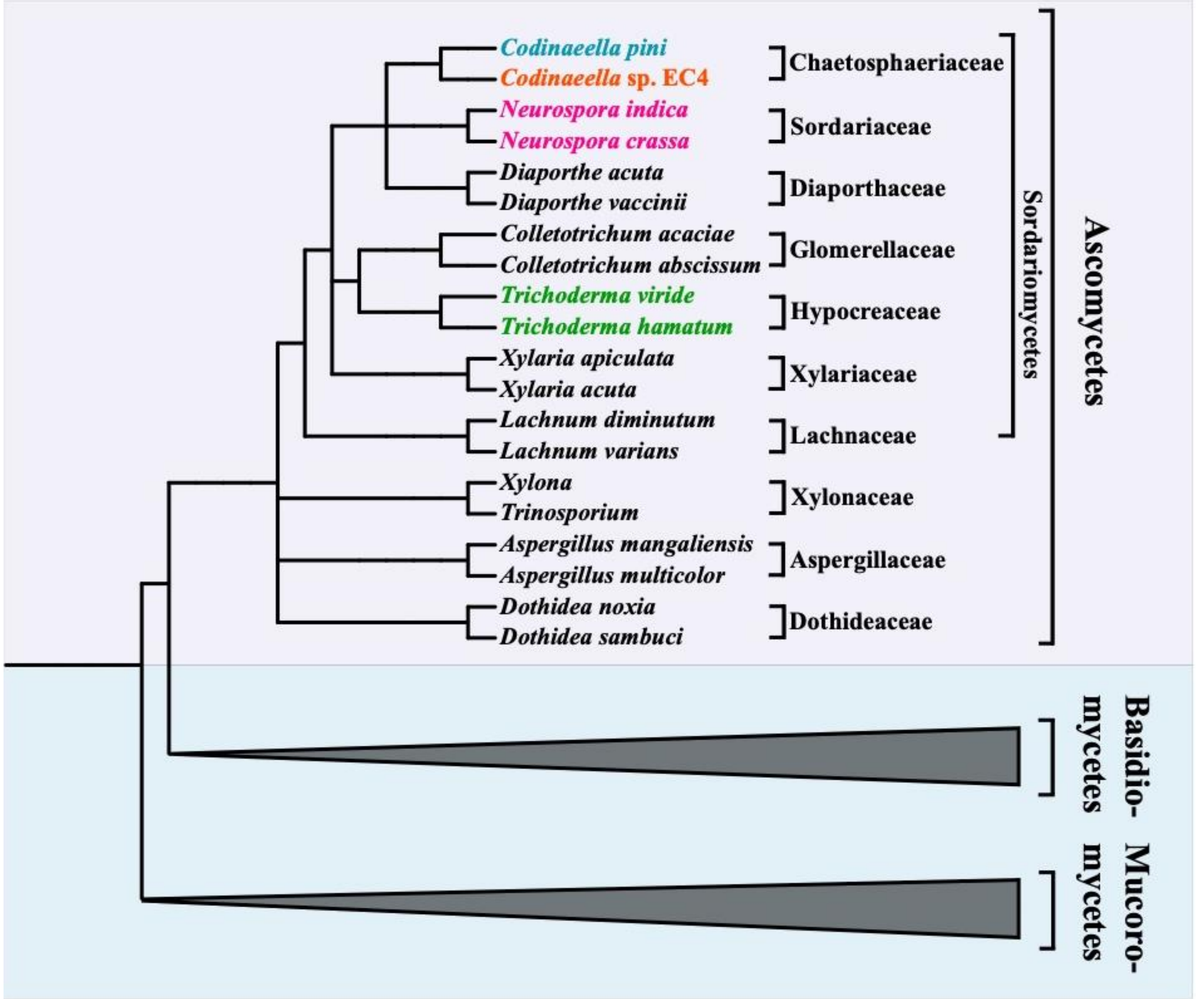

Figure S4. Schematic phylogenetic tree including EC4 and other well-studied fungi. The phylogenetic relationships between species are based on [95,96] and the tree shown in this report (Figure S2). Among the well-studied fungi, *Neurospora* and *Diaporthe* are the closest relatives of EC4.

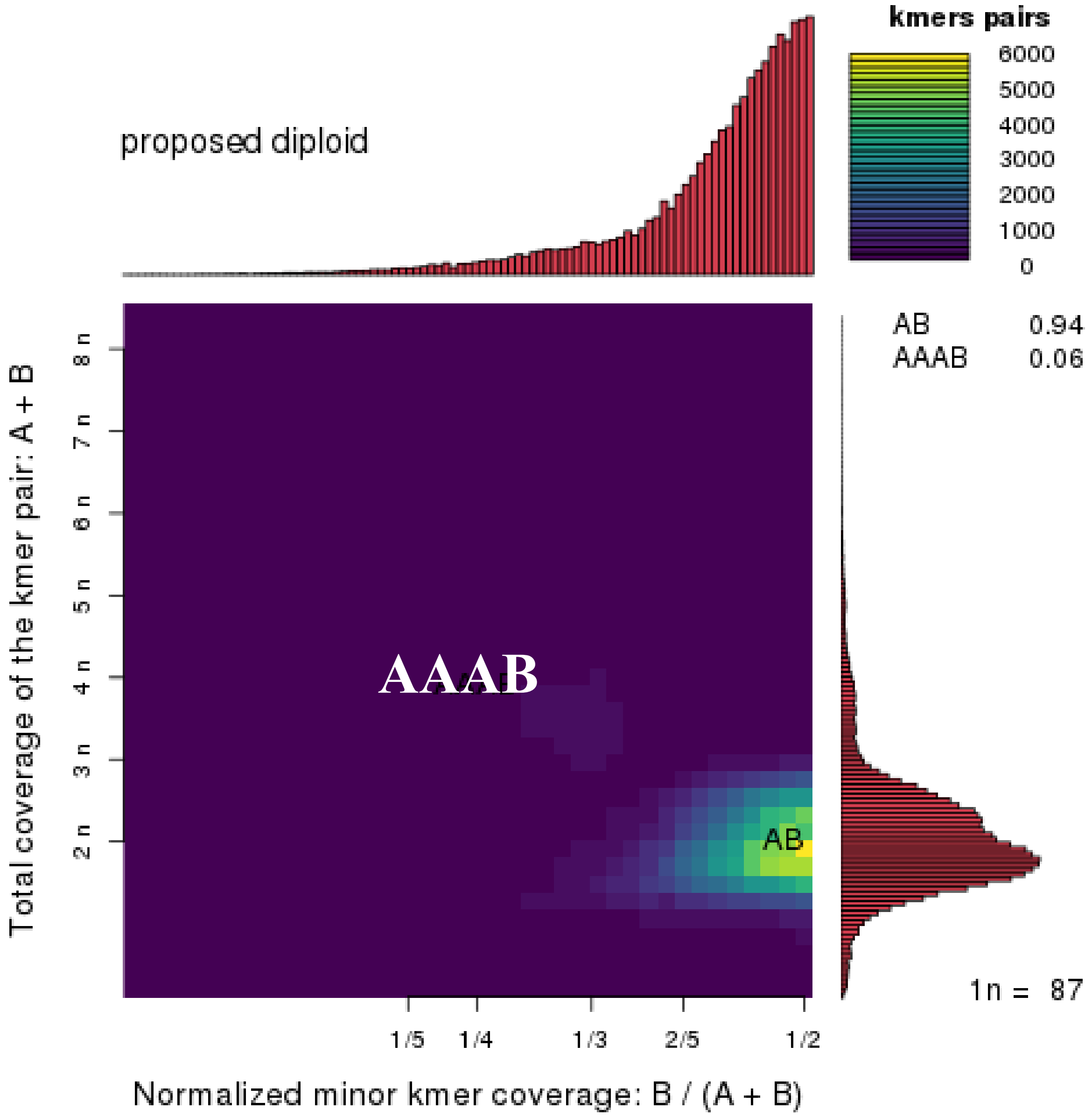

Figure S5. Ploidy inference of the EC4 nuclear genome. The ploidy estimation is based on k-mer counts in sequencing reads. The figure shows the output of Smudgeplot (v0.2.3) [44]. The heatmap (blue square) depicts the coverage of k-mer pairs differing by one nucleotide. The coverage distribution (on the right side of the heatmap) indicates the ploidy level (the scale is given on the left). The distribution on the top represents the coverage normalized by the allele ratio. EC4 is determined to be diploid. n = average k-mer coverage.

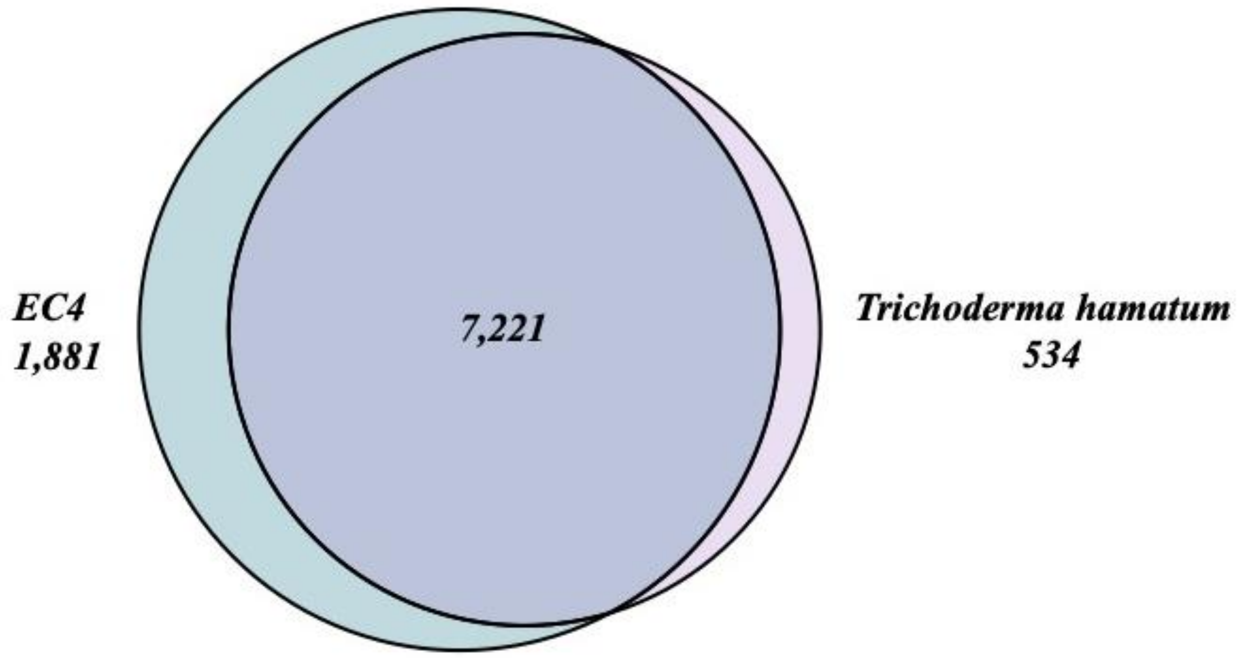

Figure S6. Analysis of orthologous genes in EC4 and *Trichoderma hamatum*. The Venn diagram represents the numbers of shared and unique gene clusters from EC4 and *Trichoderma hamatum*. The analysis was performed with Orthovenn2 [96].

Method S1. Annotation of the EC4 nuclear genome

Structural genome annotation

The structural genome annotation was performed essentially as described earlier [40]. Briefly, the genome assembly was first masked for simple repeats using RepeatScout v1.0.5 [97] and RepeatMasker v4.0.9 [unpublished: https://www.repeatmasker.org/] using the following commands:

```
build_lmer_table -sequence EC4.fasta -freq lmer_table.txt
RepeatScout -minthres 150 -sequence EC4.fasta -output repscout.fasta -freq lmer_table.txt
RepeatMasker -xsmall -gff -s -pa 40 -lib repscout.fasta EC4.fasta
```

RNA-Seq reads were quality-trimmed using triommomatic v0.30 [35], corrected for errors using Rcorrector [49], and then mapped to the genome assembly using STAR v2.6.1b [36].

```
java -jar trimmomatic-0.30.jar R1-raw.fq.gz R2-raw.fq.gz R1.fq.gz unpaired-R1.fq.gz R2.fq.gz unpaired-R2.fq.gz -phred33
ILLUMINACLIP:adapters-MI:3:30:9 LEADING:3 TRAILING:3  SLIDINGWINDOW:4:5 MINLEN:20
run_rcorrector.pl -t 20 -maxcorK 1 -k 31 R1.fq.gz R2.fq.gz
STAR --runThreadN 40 --runMode genomeGenerate --genomeDir STAR-index --genomeFastaFiles EC4.fasta --
genomeSAindexNbases 13
STAR --runThreadN 40 --genomeDir build-index --alignEndsType Local --readFilesIn R1.fq.gz R2.fq.gz --outSAMtype BAM
SortedByCoordinate --outSJfilterIntronMaxVsReadN 100 300 500 --alignIntronMin 19 --alignIntronMax 5000 --outFileNamePrefix
STAR_ --outSAMattributes All --outSAMattrIHstart 0 --outSAMstrandField intronMotif --limitBAMsortRAM 27643756136 --
readFilesCommand zcat
```

Subsequently, the processed reads were assembled by two procedures (i) *de novo* and (ii) guided by the genome assembly using Trinity v2.6.6 [98].

```
Trinity --seqType fq --max_memory 150G --left R1.fq.gz --right R2.fq.gz --CPU 40 --output trinity-denovo --full_cleanup --
SS_lib_type RF
Trinity --genome_guided_max_intron 20000 --max_memory 250G --CPU 40 --genome_guided_bam
STAR_Aligned.sortedByCoord.out.bam --output trinity-gg --full_cleanup --SS_lib_type RF
```

The resulting transcriptome assemblies were combined into a single file, trinity-comprehensive.fasta, and aligned to the genome assembly using PASA v2.3.3 [99].

```
Launch_PASA_pipeline.pl -c alignAssembly.config -C -r -R -g EC4.fasta -t trinity-comprehensive.fasta.clean -T -u trinity-
comprehensive.fasta --ALIGNERS gmap,blat --CPU 40 --TDN tdn.accs -I 20000 --stringent_alignment_overlap 30.0 –
transcribed_is_aligned_orient
```

The alignments were then combined into a single, comprehensive assembly:

```
build_comprehensive_transcriptome.dbi -c alignAssembly.config -t EC4.sqlite.assemblies.fasta --min_per_ID 95 --
min_per_aligned  95
```

Protein sequence accessions GCA_000182965.3, GCA_002759435.2, GCA_001006365.1, GCA_001702395.2, GCA_000271745.2, GCA_000002495.2, GCA_000182925.2, GCA_002072345.1, GCA_000182805.2, and GCA_001636815.1 were aligned to the genome using Spaln v2.2.2 [100].

```
spaln -C1 -O12 -Q5 -yL20 -yX -t40 -dEC4 all_protein_data.faa
```

The *ab initio* gene predictors employed were Genemark v4.33 with intron intervals as hints derived from RNA-Seq read mapping [101],

```
gmes_petap.pl --soft 1000 --ET=introns.gff --et_score=3 --cores=40 –sequence=EC4.fasta --fungus
```

CodingQuarry v2.0 was run with transcript alignments as hints [102]:

```
CodingQuarry -p 40 -f EC4.fasta -t pasa_transcripts.gff3
```

Augustus v3.3.2 [103] was employed along with protein sequence alignments, RNA-Seq read coverage and transcript alignments as described at https://bioinf.unigreifswald.de/bioinf/wiki/pmwiki.php?n=Augustus.Augustus, and Snap [104] trained on Augustus models with a score of 1 following the instructions on https://github.com/KorfLab/SNAP. Finally, the PASA assembly, Spaln alignments, and Augustus, Snap and CodingQuarry gene models were combined into a single consensus with Evidencemodeler v1.1.1 [105] following the instructions at https://evidencemodeler.github.io/. Modelling tRNAs was performed using tRNAscan-SE v1.3.1 [106]

```
tRNAscan-SE –brief –codons –output tRNAscan-SE.out EC4.fasta
```

Functional genome annotation

Coding sequences from gene models were extracted from the output of Evidencemodeler. Conceptually translated protein sequences were then searched with Blast [107] against UniProt/SwissProtKB (downloaded March 23, 2018) [108]. The GenBank [GCA_000182965.3, GCA_002759435.2,

GCA_001006365.1, GCA_001702395.2, GCA_000271745.2, GCA_000002495.2, GCA_000182925.2, GCA_002072345.1, GCA_000182805.2, and GCA_001636815.1] sequences were used for structural annotation to identify the single best hit below the maximum threshold e-value of 1.0e-7, using Blastp v2.2.31+.

```
makeblastdb –dbtype prot –in all_protein_data.faa
blastp –db all_protein_data.faa –num_threads 40 –outfmt '6 qseqid sseqid stitle pident length qlen slen evalue bitscore' –max_target_seqs 5 –evalue 1e-7 –query EC4.faa –out EC4.faa.blastp_all_protein_data
```

Product names of the single best Blast hit against the Swiss-Prot database [108] global cutoff of 1e-7) were transferred to EC4 gene models. Precedence was given to hits of GenBank accessions if the e-value was lower than a competing hit to the Swiss-Prot database; otherwise, the product name was automatically transferred in the absence of a Swiss-Prot hit, provided the e-value was below the global cutoff. We assigned 'hypothetical protein' as the product name to all remaining models without hits below the threshold. Hmmer v3.3 [38] was also used to search for conserved domains described in Pfam v31.0 using the model-specific noise threshold as the e-value cutoff. Blastp [107] and Hmmer search [49] hits were included in the 9th column of the gff3 file (https://www.ncbi.nlm.nih.gov/genbank/genomes_gff/) as 'product' and 'inference' attributes, respectively, as per the NCBI eukaryotic genome annotation guidelines (https://www.ncbi.nlm.nih.gov/genbank/eukaryotic_genome_submission_annotation/)

Result S1: Nuclear ribosomal internal transcribed spacer (ITS) sequence of endophyte re-isolated from EC4-infected cranberry plantlets

PCR with ITS PCR primers (see **Table S1**) amplified the ITS regions of EC4 and the cranberry plant. Two ITS amplicons were obtained from the root samples of EC4-inoculated plants, corresponding to the inoculated fungi EC4 and the host plant. Single ITS amplicon obtained from the root samples of not-inoculated plants corresponding to the host plant.

>Plant

**NGTNNNACNCCCNCCCGTCGCTCCTACCGATTGAATGGTCCGGTGAAGTGTTCGGATCGCGGCGACGTGGGCNGGTTCGCTGCCGGCGACGTCG
CGAGAAGTCCATGAACCTTATCATTTAGAGGAAGGAGAAGTCGTAACAAGGTTTCCGTAGGTGAACCTGCGGAAGGATCATTGTCGAAAACCTGC
CGAGCAGAAAACCCGCGAACTCGTCTCTACTCTGGGGGAACGATGCGGGGGTCGCGGCCCAGCTGCCTCGCCTTCCATTTTTTCCCCGTGCGAGC
GGATGCGCACGGAACCTCCGGGCGACGTGCTCGTCCTGCTTGTCAAACAACGAACCCCGGCGCAAGACGCGCCAAGGAAAAATCGAACAAAGAG
AGCGCGTCCCCCGCCCGTTCTCGGGCGGTGTTGGCGTCTGCAATCTTTTCTTGTAACTGAACGACTCTCGGCAACGGATATCTCGGCTCTTGCATC
GATGAAGAACGTAGCGAAATGCGATACTTGGTGTGAATTGCAGAATCCCGTGAACCATGAGTCTTTGAACGCAAGTTGCGCCTGAAGCCATTAGG
TTGAAGGCACGTCTGCCTGGGCGTCACGCATTGCGTCACCCACTCCCCCCCGCGCCCCGAGCGGGCGCGTCGGTGCGTGGGCGGATATTGGCCCC
CCGTCCGCATCCGTGCTCGGTCGGSCTAAAAAACGGGTCCCCAACGACGGACATCACGACAAGTGGTGGTTGCTAAAACCGTCGCGTCGCGTCGT
GCGTGCCATCGTCTGTTGCGGGTKGGCCCATTTGACCCTGGAGTGCCGTCTAACCGCGGCGCCTCAASTGCGACCCCNGNNCRGGGGGATTACCC
NNTGANTTTNAGCATATYNAWWNANNGGNGGNNNNN**

>EC4

**NTTTCCTCCGCTTATTGATATGCTTAAGTTCAGCGGGTATTCCTACCTGATCCGAGGTCANCCTTGTAAGATTGGGGGTTTACCGGCCGGCATGCG
CCCGCGCCCGGAACGAGAAGTAACTACTGCGTTCGGTCGTGGGACGCACCCGCCGCTGTCTTTCGGGGCCTGCAGCGCAGGACCCCAACGCCAG
GCGTGGCCTGAGGGTTGTAATGACGCTCGAACAGGCATGCCCGCCGGGATACCGGCGGGCGCAATGTGCGTTCAAAGATTCGATGATTCACTGA
ATTCTGCAATTCACATTACTTATCGCATTTCGCTGCGTTCTTCATCGATGCCAGAACCAAGAGATCCGTTGTTGAAAGTTTTAACTTATTTGTATGTA
CTCGGAGATGCCAACGCGCGAGACAGAGTATGAGGCCACCGGCGGGCGTAGCGCCCCGGACGAACCGGAGCACCCGCCGAGGCAACGACTATA
GGTATGTTCACAGGGGTTACGGGAGTCTTTCGAGTCCTGTAATGATCCCTCCGCTGGTTCACCAACGGAGACCTTGTTACGACTTTTACTTCCTCTA
AATGACCGAGTTTGGATAGCTTTCCGGCCCTGGGTGGCCGTTGCCGGCCTCCCTGGGCCAGTCCGGAAGCCTCACTGAGCCATTCAATCGGTAGT
AGCGACGGGCGGNGTGTACN**
